# Effects of a high-sodium diet on renal tubule Ca^2+^ transporter and claudin expression in Wistar-Kyoto rats

**DOI:** 10.1186/1471-2369-13-160

**Published:** 2012-12-02

**Authors:** Midori Sasaki Yatabe, Junichi Yatabe, Kozue Takano, Yuta Murakami, Rina Sakuta, Sadahiko Abe, Hironobu Sanada, Junko Kimura, Tsuyoshi Watanabe

**Affiliations:** 1 Department of Pharmacology, Fukushima Medical University School of Medicine, 1 Hikarigaoka, Fukushima, 960-1295, Japan; 2 Department of Nephrology, Hypertension, Diabetology, Endocrinology and Metabolism, Fukushima Medical University School of Medicine, Fukushima, Japan; 3 Division of Health Science Research, Fukushima Welfare Federation of Agricultural Cooperatives, Fukushima, Japan

**Keywords:** Calcium, Sodium chloride, Distal tubule, Na^+^/Ca^2+^ exchanger, Ca^2+^ channel, Claudins

## Abstract

**Background:**

Urinary Ca^2+^ excretion increases with dietary NaCl. NaCl-induced calciuria may be associated with hypertension, urinary stone formation and osteoporosis, but its mechanism and long-term effects are not fully understood. This study examined alterations in the expressions of renal Ca^2+^ transporters, channels and claudins upon salt loading to better understand the mechanism of salt-induced urinary Ca^2+^ loss.

**Methods:**

Eight-week old Wistar-Kyoto rats were fed either 0.3% or 8% NaCl diet for 8 weeks. Renal cortical expressions of Na^+^/Ca^2+^ exchanger 1 (NCX1), Ca^2+^ pump (PCMA1b), Ca^2+^ channel (TRPV5), calbindin-D_28k,_ and claudins (CLDN-2, -7, -8, -16 and −19) were analyzed by quantitative PCR, western blot and/or immunohistochemistry.

**Results:**

Fractional excretion of Ca^2+^ increased 6.0 fold with high-salt diet. Renal cortical claudin-2 protein decreased by approximately 20% with decreased immunological staining on tissue sections. Claudin-16 and −19 expressions were not altered. Renal cortical TRPV5, calbindin-D_28k_ and NCX1 expressions increased 1.6, 1.5 and 1.2 fold, respectively.

**Conclusions:**

Chronic high-salt diet decreased claudin-2 protein and increased renal TRPV5, calbindin-D_28k_, and NCX1. Salt loading is known to reduce the proximal tubular reabsorption of both Na^+^ and Ca^2+^. The reduction in claudin-2 protein expression may be partly responsible for the reduced Ca^2+^ reabsorption in this segment. The concerted upregulation of more distal Ca^2+^-transporting molecules may be a physiological response to curtail the loss of Ca^2+^, although the magnitude of compensation does not seem adequate to bring the urinary Ca^2+^ excretion down to that of the normal-diet group.

## Background

Urinary Ca^2+^ excretion increases with sodium chloride (NaCl) ingestion [1]. This dietary NaCl-induced calciuria may lead to osteoporosis at low calcium intake [2,3] and also is associated with urinary stone formation [1] and hypertension [4]. The increase in urinary Ca^2+^ excretion is postulated to be due to salt-induced volume expansion [5] and/or competition between sodium and calcium ions in the renal tubule [6]. However, the precise mechanism for the dietary NaCl-induced urinary Ca^2+^ increase is not fully understood. In addition, it is not clear if long-term salt loading has any effects on Ca^2+^-transporting molecule expressions in the kidney.

The bulk of Ca^2+^ in the pro-urine is reabsorbed in the proximal tubule and the thick ascending loop of Henle through a passive, paracellular movement. Transepithelial Ca^2+^ permeability is high in these segments, and the rate-limiting barrier is the tight junction. Claudins and other tight junction proteins are known to be important in determining the permeability characteristics of various epithelia [7]. For example, renal expression of claudin 2 is restricted to the proximal nephron [8], and claudin 2 is believed to form high-conductance cation pores [9]. The distributions and functions of these tight junction proteins are becoming known, but information on their regulation, especially in the kidney, is just emerging.

In contrast, regulated transcellular Ca^2+^ reabsorption occurs primarily in the distal tubule. In the distal nephron, Ca^2+^ in the pro-urine enters the cytosol of tubule cells through Ca^2+^ channel, mainly TRPV5 [10]. The transport of intracellular Ca^2+^ to the basolateral side is facilitated by a Ca^2+^-binding protein called calbindin-D_28k_[10,11], and Ca^2+^ exits the cell on the basolateral side through Na^+/^Ca^2+^ exchanger 1 (NCX1) and Ca^2+^ pump (PMCA1b) [12,13]. NCX1 counter-transports 3 Na^+^ for Ca^2+^, but the role of NCX1 in NaCl-induced calciuria has not been studied.

Alterations in the expressions of tight junction proteins and transcellular Ca^2+^ transporters may in part explain the urinary calcium loss upon salt loading or provide clues on long-term effects of dietary NaCl ingestion. Therefore, we examined the expression changes of renal Ca^2+^ transport molecules in rat with chronic high-NaCl diet.

## Methods

### Animal experiment

All experimental procedures were approved by the Fukushima Medical University School of Medicine Animal Committee. Eight-week old Wistar-Kyoto rats (Japan SLC Inc. Sendai, Japan) were fed either 0.3% or 8% NaCl chow (Oriental Yeast Co., Tokyo, Japan) for 8 weeks with tap water ad libitum. Unanesthetized systolic blood pressure was measured by the tail-cuff method (Blood Pressure Analyzer model BP-98A; Softron, Tokyo, Japan). Ten measurements were taken and averaged per rat per day. Urine was collected regularly using metabolic cages. At the end of the study, under intraperitoneal pentobarbital anesthesia, blood was drawn from abdominal aorta, and kidneys were collected for assays.

### Biochemical analysis

Biochemical analyses were performed by SRL Inc. (Tokyo, Japan) using creatinase-sarcosine-oxidase-POD method for creatinine, electrode method for Na, K and Cl, arsenazo III method for Ca, direct molybdate assay for inorganic phosphate (P) and xylidyl blue method for Mg. Serum concentrations of 1,25-dihydroxyvitamin D_3_ were measured by radioimmunoassay using the two antibody method.

### Quantitative Real-Time RT-PCR

Total RNA was prepared from renal cortex using RNeasy plus mini kit (Qiagen). Subsequently, 0.25 μg of total RNA was reverse-transcribed into cDNA using iScript cDNA Synthesis Kit (Bio Rad) in a 20 μl reaction volume. One μl of reverse-transcription sample was used for real-time quantitative PCR using the iQ5 Real-Time PCR Detection System and iQ SYBR Green Supermix (Bio Rad). The primers used were as follows: NCX1 forward CAGTTGTGTTTGTCGCTCTTGG and reverse GTTGGCCGCATGGTAGATGG, with annealing temperature (Ta) 57°C; GAPDH forward GCAAGTTCAACGGCACAGTCAAG and reverse ACATACTCAGCACCAGCATCACC with Ta 56°C, TRPV5 forward CTTACGGGTTGAACACCACCA and reverse TTGCAGAACCACAGAGCCTCTA with Ta 56°C; PMCA1b forward CGCCATCTTCTGCACAATT and reverse CAGCCATTGTTCTATTGAAAGTTC with Ta 56°C, calbindin-D_28k_ forward GGAGCTGCAGAACTTGATCCand reverse GCAGCAGGAAATTCTCTTCG with Ta 57°C, claudin 2 forward TCTGGATGGAGTGTGCGAC and reverse AGTGGCAAGAGGCTGGGC with Ta 63°C, claudin 7 forward GACTCGGTGCTTGCCCTGCC and reverse GGAGCGGGGTGCACGGTATG with Ta 59°C, claudin 8 forward GTGCTGCGTCCGTCCTGTCC and reverse CCAAGCTCGCGCTTTTGGGC with Ta 59°C. NCX1 and GAPDH primers were designed using Beacon Designer software (PREMIER Biosoft International, Palo Alto, California, USA), claudin 7 and 8 primers were designed using PrimerBlast (NCBI), and TRPV5, PMCA1b and claudin 2, 16 and 19 primers were adopted from elsewhere [14-16]. PCR reactions were performed in triplicate, and mRNA was quantified based on the Ct value, normalized to GAPDH, and expressed as relative amounts.

### Immunoblotting

Immunoblotting of renal cortical proteins was performed similarly as previously reported [17]. The antibodies used were monoclonal anti-rat NCX1 antibody (Abcam), polyclonal anti-claudin 2 antibody (Life Technologies, Carlsbad, CA), and polyclonal anti-TRPV5, anti-NHE3, and anti-GAPDH antibodies (Santa Cruz Biotechnology). The bands were visualized by ECL or ECL plus reagents (Amersham) and quantified by densitometry using ImageJ software.

### Immunohistochemistry

Sections of rat kidney paraffin blocks were made with 2-μm thickness. Kidney sections of normal- and high-salt diet fed rats were placed on a single slide glass for comparison. After deparaffinization and blocking, the slices were treated with anti-claudin 2 antibody (Life Technologies, Carlsbad, CA), anti-rabbit secondary antibody and DAB using VECTASTAIN-ABC kit (Vector laboratories, Burlingame, CA). The slides were counterstained with haematoxylin and eosin.

### Data analysis

All values are expressed as means ± SE. Statistical comparisons were performed by Student's *t*-test or ANOVA where appropriate. *P* values <0.05 were considered statistically significant.

## Results

### Serum electrolytes were similar between the normal- and high-salt fed rats

**Figure 1:**
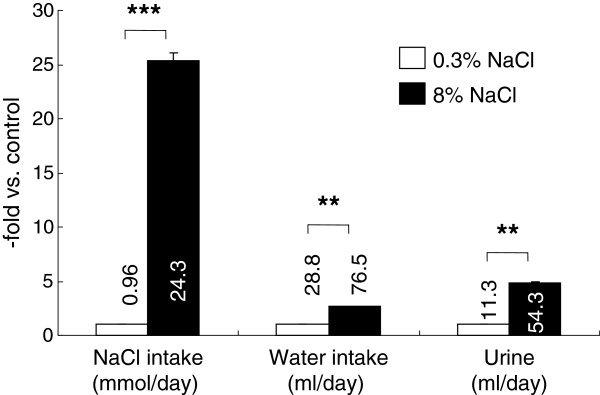
**Intake and output data at the end of the study in Wistar-Kyoto rats fed 0.3% or 8% NaCl diet for 8 weeks.** The bars are shown as relative amounts of the 8% NaCl group to those of 0.3% NaCl control group. Numbers by the bars are the averages of actual measurements, with the units indicated below the item labels. * *P* <0.05, ** *P* <0.01, and *** *P* <0.001 vs. 0.3% NaCl group of same age, n=15.

Food intake was similar between the groups (normal-salt vs high-salt groups, 18.8±0.9 vs 17.8±0.5 g/day, n.s., n=15 /group), although the high-salt group weighed slightly less than the normal-salt group at the end of the study (369±5 vs 354±4 g, *P* <0.05). This may partly be due to the reduced caloric intake of the high-salt fed rats because 8% (by weight) of the chow was sodium chloride. As expected, the high-salt group drank and urinated significantly more than the normal-salt group, 2.7 times and 4.8 times the control rats, respectively (Figure 1). However, the serum electrolyte concentrations measured did not differ between the normal-salt and high-salt groups (Table 1). Creatinine clearance, which is used as an approximate of glomerular filtration rate, was also not significantly different between the groups (2.51±0.12 vs 2.63±0.14 ml/min, n=12-15 /group). Systolic blood pressures also did not differ significantly between the groups (133±2.8 vs 141±3.0 mmHg, n=15/group).

**Table 1:** Blood pressure and biochemistry data at the end of the study

	**0.3% NaCl**			**8% NaCl**			
Systolic Blood Pressure (mmHg)	133	±	2.8	141	±	3	n.s.
Serum creatinine (mg/dL)	0.29	±	0.01	0.33	±	0.02	n.s.
Serum Na (mmol/L)	143	±	0.5	142	±	0.3	n.s.
Serum K (mmol/L)	4.33	±	0.07	4.36	±	0.11	n.s.
Serum Cl (mmol/L)	104	±	0.69	103	±	0.64	n.s.
Serum Ca (mmol/L)	9.26	±	0.07	9.22	±	0.09	n.s.
Serum Mg (mmol/L)	2.29	±	0.03	2.23	±	0.04	n.s.
Serum P (mmol/L)	7.05	±	0.17	7.06	±	0.23	n.s.

Abbreviations: *n.s.*: not significant.

### Urinary calcium excretion was markedly increased in the high-salt rats

**Figure 2:**
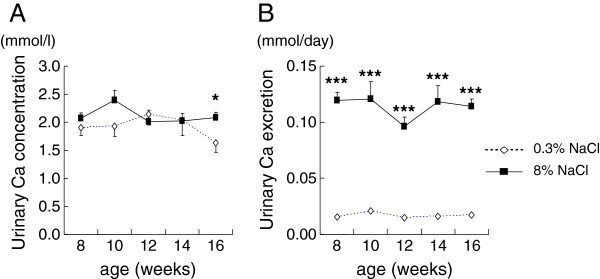
**Urinary calcium concentration (2A) and daily urinary calcium excretion (2B) of rats fed normal-salt or high-salt diet from 8 to 16 weeks of age.** * *P* <0.05 and *** *P* <0.001 vs. 0.3% NaCl group of same age, n=5-15.

**Figure 3:**
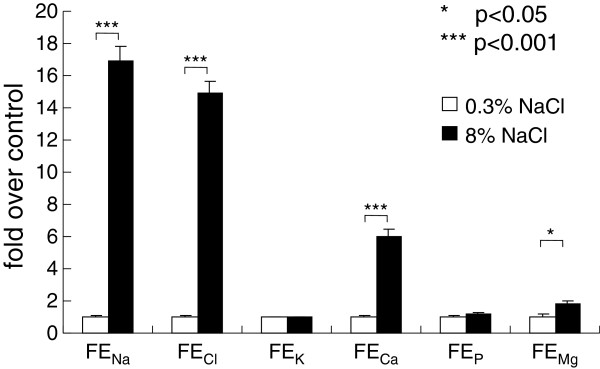
**Fractional excretion of electrolytes at the end of the study.** The bars are shown as relative amounts of the 8% NaCl group to those of 0.3% NaCl control group. * *P* <0.05 and *** *P* <0.001 vs. 0.3% NaCl group of same age, n=15.

At the end of the study, urinary calcium concentration (Figure 2A) and daily urinary calcium excretion (Figure 2B) of rats on high-salt diet were higher than those of the normal-salt group, and fractional Ca excretion of the salt-loaded rats was 6 times that of the control rats (Figure 3). Fractional Mg excretion also increased with salt loading, although the increase was smaller than that of the fractional Ca excretion (Figure 3).

### Renal claudin-2 protein decreased, but claudin-7, -8, -16 or -19 mRNA was not altered with chronic salt loading

**Figure 4:**
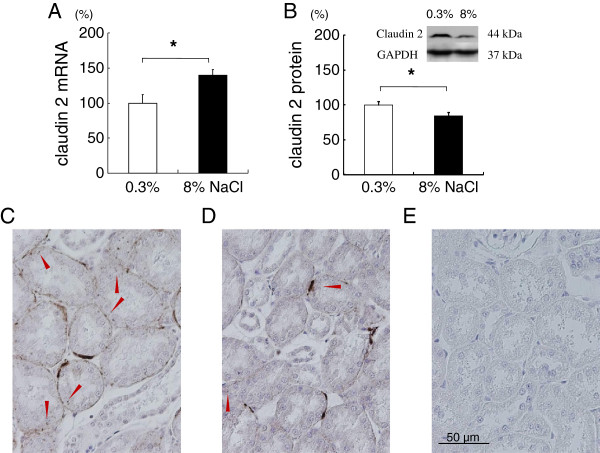
**Claudin-2 expressions in the kidney cortex of rats fed 0.3% or 8% NaCl for 8 weeks.** Protein and mRNA were normalized against GAPDH expression and expressed as relative amounts. Salt loading for 8 weeks increased the expression of claudin-2 mRNA (**4A**), but significantly decreased its protein level (**4B**). * *P* <0.05 vs 0.3% NaCl group, n=14-16. Claudin 2 immunnohistochemistry in kidney from rat on normal-salt diet (**4C**), high-salt diet (**4D**), and no primary antibody control (**4E**). Arrowheads indicate claudin 2 staining. By subjective observation, normal-salt fed rat kidney (**4C**) shows more claudin 2 staining compared to salt-loaded rat kidney (**4D**).

Claudin-2 forms paracellular cation pore in the proximal tubule. Rats fed 8% NaCl for 8 weeks showed increased renal cortical claudin-2 mRNA (Figure 4A), but salt loading significantly decreased the protein expression of claudin-2 by about 20% (Figure 4B). There may be post-transcriptional regulation of claudin-2. Immunohistochemical staining of kidney cortex was performed for claudin-2 to further examine the change in expression. Although by subjective observation, the staining of renal cortical claudin-2 also suggested a decrease by salt loading (Figure 4C and 4D). In the proximal tubule, NHE3 expressed primarily in the apical membrane is shown to be necessary for calcium reabsorption by providing the driving force for paracellular calcium transport [18]. Unexpectedly, this study found that renal cortical NHE3 protein level of salt-loaded rats was significantly increased compared to that of rats on normal diet (100±20 vs 292±38%, n=9-11, P<0.01, figure not shown).

**Figure 5:**
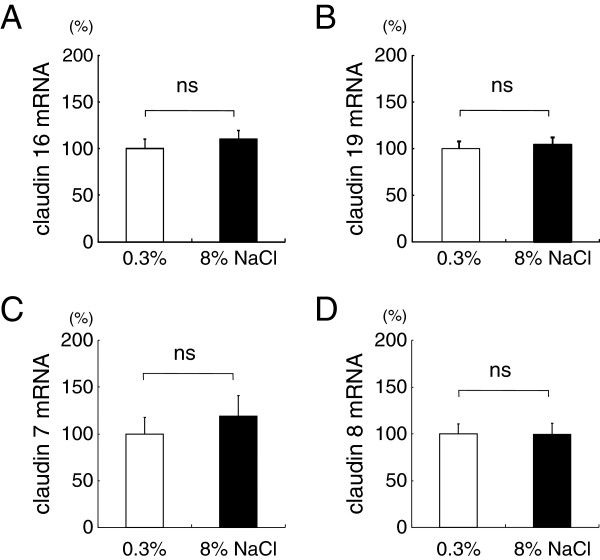
**Claudin-16, -19, -7, and −8 expressions in the kidney cortex of rats fed 0.3% or 8% NaCl for 8 weeks.** Target mRNA or protein levels were normalized against GAPDH and expressed as relative amounts. Salt loading for 8 weeks did not significantly alter claudin-16 (**5A**), -19 (**5B**), -7 (**5C**), or −8 (**5D**) mRNA. n=13-15 for **5A** and **5B**, and n=9-10 for **5C** and **5D**.

Claudin-16 and −19 are expressed primarily in the thick ascending limb [19], and mutations of claudin-16 [20] and −19 [21] result in renal Mg^2+^ and Ca^2+^ wasting. In this study, no significant change in renal claudin-16 or −19 was observed by salt loading (Figure 5A and 5B).

Claudin-7 and −8 are found from distal convoluted tubule to the inner medullary collecting duct [22]. Claudin-8 is believed to act as a paracellular cation barrier [23], inhibiting the backflow of Ca^2+^ that has been reabsorbed through transcellular mechanisms. Claudin-7 is generally assumed to be an anion barrier. No significant change was observed in the mRNA expression of claudin-7 or claudin-8 (Figure 5C and 5D).

*High-salt diet increased distal, transcellular* Ca^2+^ transporting molecules, *TRPV5, calbindin-D*_*28*_*, and NCX1*.

**Figure 6:**
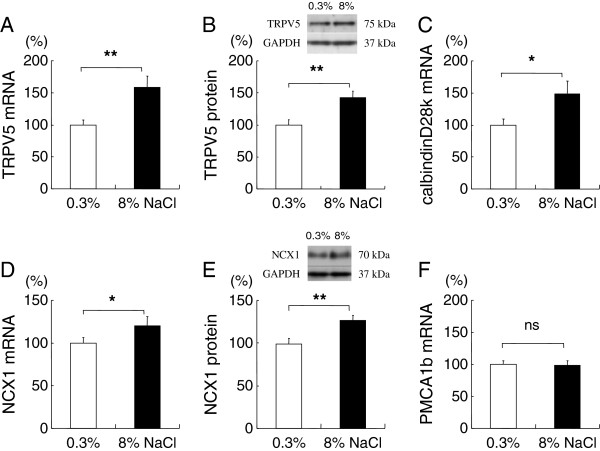
**Distal, transcellular calcium-transporting molecule expressions in the kidney cortex of rats fed 0.3% or 8% NaCl for 8 weeks.** Protein and mRNA of the target molecules were normalized against GAPDH expression and expressed as relative amounts. Salt loading increased the expression levels of TRPV5 (**6A**: mRNA, **6B**: protein), calbindin-D_28k_ (**6C**), and NCX1 (**6D**: mRNA, **6E**: protein), but did not alter PMCA1b (**6F**) expression. * *P* <0.05 and ** *P* <0.01, vs. 0.3% NaCl group, n=14-16.

TRPV5 Ca^2+^ expression in the renal cortex increased with high-salt diet both in terms of mRNA (Figure 6A) and protein levels (Figure 6B). TRPV5 is the apical Ca^2+^ entry mechanism and the gatekeeper of the distal tubular Ca^2+^ transport [24]. In addition, the renal cortical mRNA of calbindin-D_28k_, an intracellular Ca^2+^ transport molecule [11], also increased by about 48% (Figure 6C). NCX1 and PMCA1b are the basolateral Ca^2+^ extrusion mechanisms in this segment [25]. Renal cortical NCX1 mRNA and protein levels increased in the high-salt group by about 20% and 26%, respectively (Figure 6D and E). In contrast, renal cortical expression of PMCA1b was not altered by high-salt diet (Figure 6F).

### Modeling of NCX1 function

**Figure 7:**
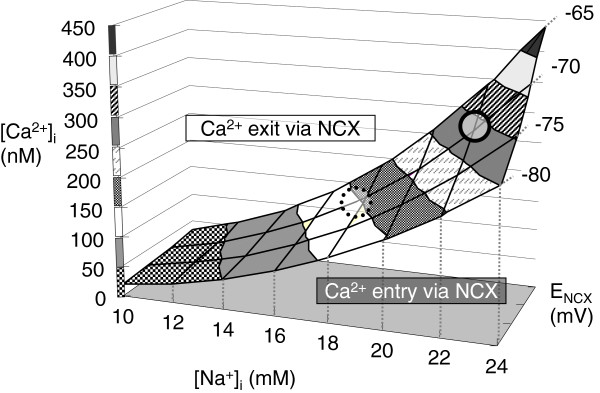
**Simulated E**_**NCX**_**, [Na**^**+**^**]**_**i**_**and [Ca**^**2+**^**]**_**i**_**, assuming [Na**^**+**^**]**_**o**_**= 140 mM and [Ca**^**2+**^**]**_**o**_**= 1 mM.** Abbreviations; E_NCX_: equilibrium potential of NCX1, [Na^+^]_i_ : intracellular Na^+^ concentration, [Ca^2+^]_i_: intracellular Ca^2+^ concentration, [Na^+^]_o_: extracellular (basolateral) Na^+^ concentration and [Ca^2+^]_o_: extracellular (basolateral) Ca^2+^ concentration. Dotted circle indicates the simulated physiological [Ca^2+^]_i_, with [Na^+^]_i_ at 17.5 mM and E_NCX_ at −70 mV. Solid circle indicates the simulated [Ca^2+^]_i_ with salt loading, with [Na^+^]_i_ assumed at 22 mM and E_NCX_ at −70 mV.

The functions of NCX1 with different electrolyte settings were modeled to interpret the effects of high-salt diet because the transport of NCX1 is bidirectional. Even under normal conditions, estimates of NCX1 contribution to distal tubular, basolateral Ca^2+^ transport vary from 15% [26] to 70% [27]. Figure 7 shows the relationship among intracellular Na^+^ concentration ([Na^+^_i_), intracellular Ca^2+^ concentration [Ca^2+^_i_, and equilibrium potential for NCX (E_NCX_), calculated using the equation, E_NCX_ = 3 E_Na_ - 2 E_Ca_[28], where E_Na_ and E_Ca_ are respective equilibrium potentials for Na^+^ and Ca^2+^ given by the Nernst equation. Basolateral extracellular Na^+^ concentration is set at 140 mM and basolateral extracellular Ca^2+^ concentration at 1 mM. In the literature, the basolateral membrane potential of the distal convoluted tubule and connecting tubule cells is reported to be −70 mV [29], while [Na^+^_i_ in those cells is reported to be 17.5 mM [26,30]. Under these conditions, the model in Figure 7 gives [Ca^2+^_i_ of 142 nM (shown as a dotted circle). This [Ca^2+^_i_ is below the estimated [Ca^2+^_i_ of 200 nM [29], indicating that NCX1 likely extrudes Ca^2+^ under normal conditions. However, the effects of high-salt diet on Na^+^ and Ca^2+^ gradients and membrane potential have not been determined. As dietary NaCl reduces plasma aldosterone and increases endogenous Na^+^/K^+^ pump inhibitors such as ouabain [31] and marinobufagenin [32,33], [Na^+^_i_ is likely to be elevated. If [Na^+^_i_ rises to 22 mM at -70mV, the equilibrium [Ca^2+^_i_ will be 282 nM (shown as a solid circle on Figure 7). Then, NCX1 extrudes less Ca^2+^ from the cell, or may even reverse to Ca^2+^ entry mode on high-salt diet. As TRPV5 is inhibited by a rise in [Ca^2+^_I_[34], the elevation of intracellular [Ca^2+^_i_ along with [Na^+^_i_ may reduce the rate of Ca^2+^ entry via TRPV5.

### Chronic salt loading decreased serum 1,25-dihydroxyvitamin D_3_ concentration

To investigate the mechanism of upregulation of TRPV5, calbindin-D_28k_ and NCX1 with salt loading, serum concentrations of 1,25-dihydroxyvitamin D_3_ [1,25(OH)_2_D] were measured. 1,25(OH)_2_D is known to upregulate renal TRPV5, calbindin-D_28k_ and NCX1 [35]. However, rats fed 8% NaCl diet for 8 weeks showed significantly reduced serum concentration of 1,25(OH)_2_D (176±19 vs 129±7 pg/ml, *P* <0.05, n=9-11), suggesting the presence of mechanisms other than 1,25(OH)_2_D for the salt-induced upregulation of these molecules.

## Discussion

This study, for the first time, examined the effects of long-term dietary sodium chloride on renal Ca^2+^-transporting molecule and claudin expressions. Chronic salt loading decreased the protein expression of claudin 2, a component of proximal, paracellular Ca^2+^ transport pathway. Concomitantly, dietary NaCl increased the expression of more distal, transcellular Ca^2+^ reabsorption machinery, TRPV5, calbindin-D_28k_ and NCX1.

Salt loading acutely increases urinary excretion of Ca^2+^ along with Na^+^[3,6]. In this study, the fractional Ca^2+^ excretion of salt-loaded rats increased approximately 6.0 fold. Generally, the cause for this phenomenon is attributed to an extracellular fluid volume expansion and/or to the reduced reabsorption of both Na^+^ and Ca^2+^ in the proximal tubule [36]. Although renal blood flow is reported to be unchanged or sometimes even reduced when salt loading is chronic such as over 8 weeks [37], from this study, the contribution of volume expansion and/or hyperfiltration cannot be ruled out as creatinine clearance tended to increase in the salt-loaded rats, although not significant. As creatinine determination in rodents can vary depending on the method used [38], the use of inulin clearance may be favorable. Pressure natriuresis is another possible factor of salt-induced calciuria, as blood pressure of salt-loaded rats tended to increase, although the difference was not statistically significant. Renal artery servo-control experiments would be useful to delineate these in the future.

Approximately 65% of calcium in the pro-urine is reabsorbed in the proximal tubule. In the proximal tubule, claudin-2 is postulated to form tight junction cation pores [9]. Muto et al. have reported that fractional excretion of Ca^2+^ in claudin-2 knockout mice is 3 times that of wild-type mice, further supporting a role of claudin-2 in proximal tubular paracellular Ca^2+^ reabsorption [39]. In this study, we found that chronic salt loading decreased renal cortical claudin-2 protein expression. Although there is not enough functional studies of rat claudin-2, sequence similarity to mouse claudin-2 suggests a similar role in Na^+^ and Ca^2+^ transport. Therefore, the decreased expression of claudin-2 with high-salt diet may, to some degree, account for the decrease in Ca^2+^ reabsorption, while limiting Na^+^ and water reabsorption, as Na^+^[9] and water [40] in addition to Ca^2+^ may pass through the pores formed by claudin-2. It has been reported that hyperosmolarity stress decreased claudin-2 expression in Madin-Darby canine kidney cells [41], and hyperosmolarity due to NaCl load may be a possible mechanism of claudin-2 downregulation in this study. As claudin-2 facilitates Ca^2+^ movement from the luminal to interstitial fluid in the proximal tubule, reduction in claudin-2 may underlie the increased urinary Ca^2+^ excretion observed under high-salt diet.

In the proximal tubule, NHE3 is shown to be important as a part of driving force for Ca^2+^ reabsorption, mediating apical Na^+^ entry and consequently water reabsorption to produce osmotic gradient [18]. In our study, renal NHE3 protein significantly increased with salt loading. However, this finding is not in accordance with some previous studies, such as that of Frindt and Palmer who found no change in luminal NHE3 with 5% NaCl diet for 1 week in rats using in situ biotinylation [42]. As regulation of NHE3 occurs on multiple levels, including trafficking, interacting proteins and oligomerization [43], protein level may not be directly related to apical NHE3 activity. If NHE3 activity is indeed increased in the high salt-fed rats, this may increase the pressure for Ca^2+^ reabsorption in the proximal tubule. However, competition between Na^+^ and Ca^2+^ for paracellular transport binding site may occur in the proximal tubule. It has been reported that Ca^2+^ inhibits paracellular Na^+^ conductance by competitive binding on claudin-2 [44]. If Na^+^ and Ca^2+^ share a binding site, inversely, high Na^+^ may inhibit claudin-2 Ca^2+^ conductance. This competition between Na^+^ and Ca^2+^ may play a large role in the dietary NaCl-induced hypercalciuria.

Thick ascending limb of the loop of Henle is responsible for approximately 20% of Ca^2+^ reabsorption. Claudin-16 and −19 are shown to be important for paracellular Mg^2+^ and Ca^2+^ in this segment. In our study, there was an increase in the fractional excretion of Mg, albeit smaller than that of Ca. However, there was no significant difference in renal claudin-16 or −19 mRNA in rats on high-salt diet. Extracellular volume expansion decreases transepithelial voltage and Mg^2+^ reabsorption in the TAL [45]. Although not directly detectable in our experimental setting, there may have been some volume expansion in the high-salt fed rats which may have contributed to the increase in Mg^2+^ fractional excretion.

Distal nephron is the final and most-regulated site of urinary Ca^2+^ reabsorption [46,47]. A concerted increase in the expression levels of TRPV5, calbindin-D_28k_, and NCX1, was observed with salt loading in this study. Claudin-8, the distal tubular paracellular cation barrier, was not altered by salt loading. It may be that with salt loading, the proximal, paracellular Ca^2+^ reabsorption is reduced, and more distal, transcellular Ca^2+^ transport molecules are upregulated to facilitate Ca^2+^ reabsorption as a compensatory mechanism. However, salt loading may reduce the Ca^2+^ reabsorption via NCX1, as illustrated in Figure 7. Therefore, the upregulation of distal Ca^2+^ transport machinery with chronic salt-loading may partially compensate for the urinary Ca^2+^ loss, although with a limited effect.

As for the mechanism of TRPV5, calbindin-D_28k_, and NCX1 upregulations by dietary NaCl, one possibility is the endocrine factors that regulate Ca^2+^-related molecules, such as parathyroid hormone [48] and vitamin D [49]. For example, 1,25(OH)_2_D has been shown to increase the expressions of TRPV5, calbindinD_28k_, and NCX1 [35]. However, in this study, serum concentration of 1,25(OH)_2_D was significantly lower in the high-salt group than the control group. Unless there is a significant difference between serum and intrarenal 1,25(OH)_2_D levels, it is likely that salt-induced transcellular Ca^2+^-transporter upregulation is mediated by pathway(s) other than 1,25(OH)_2_D.

The weakness of the study includes a lack of regional expression data, as excised renal cortex was used in the study. Higher-resolution immunohistological staining experiments and qRT-PCR/Western blotting from micro-dissected tissue specimens are necessary in the future. However, this study aimed to lay the foundation for a more detailed mechanistic examination of the effects of chronically high dietary sodium on the expression of renal Ca transporters and on urinary calcium excretion.

## Conclusions

Our findings suggest that the decrease in renal claudin-2 protein by salt loading may increase the Ca^2+^ in tubular fluid reaching the distal tubule, while the concerted upregulation of more distal Ca^2+^-handling molecules may curtail some of the Ca^2+^ loss in the urine. Findings of our study may have implications on further research on the pathophysiology of osteoporosis, urinary stone formation and hypertension associated with excessive salt intake.

## Abbreviations

NCX1: Na^+^/Ca^2+^ exchanger 1; 1,25(OH)_2_D: 1,25-dihydroxyvitamin D_3_.

## Competing interests

The authors declare that they have no competing interests.

## Authors’ contributions

MSY conceived the experiments in part with JY. KT, YM, RS and SA performed the experiments. HS, JK and TW gave advice on the study. All authors read and approved the final manuscript.

## Pre-publication history

The pre-publication history for this paper can be accessed here:

http://www.biomedcentral.com/1471-2369/13/160/prepub
